# The Positive and Negative Syndrome Scale (PANSS): A Three-Factor Model of Psychopathology in Marginally Housed Persons with Substance Dependence and Psychiatric Illness

**DOI:** 10.1371/journal.pone.0151648

**Published:** 2016-03-21

**Authors:** Chantelle J. Giesbrecht, Norm O’Rourke, Olga Leonova, Verena Strehlau, Karine Paquet, Fidel Vila-Rodriguez, William J. Panenka, G. William MacEwan, Geoffrey N. Smith, Allen E. Thornton, William G. Honer

**Affiliations:** 1 Department of Psychology, Simon Fraser University, Burnaby, British Columbia, Canada; 2 Department of Public Health and Center for Multidisciplinary Research in Aging, Ben-Gurion University of the Negev, Be’er Sheva, Israel; 3 Department of Psychiatry, University of British Columbia, Vancouver, British Columbia, Canada; University of Granada, SPAIN

## Abstract

Rates of psychopathology are elevated in marginalized and unstably housed persons, underscoring the need for applicable clinical measures for these populations. The Positive and Negative Syndrome Scale (PANSS) is a clinical instrument principally developed for use in schizophrenia to identify the presence and severity of psychopathology symptoms. The current study investigates whether a reliable and valid PANSS factor structure emerges in a marginally housed, heterogeneous sample recruited from the Downtown Eastside of Vancouver where substance use disorders and psychiatric illness are pervasive. Participants (n = 270) underwent structured clinical assessments including the PANSS and then were randomly assigned to either exploratory (EFA) or confirmatory factor analytic (CFA) subsamples. EFA pointed to a novel three factor PANSS. This solution was supported by CFA. All retained items (28 out of 30) load significantly upon hypothesized factors and model goodness of fit analyses are in the acceptable to good range. Each of the three first-order factor constructs, labeled Psychosis/Disorganized, Negative Symptoms/Hostility, and Insight/Awareness, contributed significantly to measurement of a higher-order psychopathology construct. Further, the latent structure of this 3-factor solution appears temporally consistent over one-year. This PANSS factor structure appears valid and reliable for use in persons with multimorbidity, including substance use disorders. The structure is somewhat distinct from existing solutions likely due to the unique characteristics of this marginally housed sample.

## Introduction

Persons living in Single-Room Occupancy Hotels (SROH) are socially and economically marginalized. They experience transient housing and occasional episodes of absolute homelessness [1]. In the SROHs located on the Downtown Eastside (DTES) of Vancouver, Canada rates of psychosis approach 50% [2], a proportion that is consistent with that reported in other urban communities [3,4]. Further, in Vancouver’s SROHs polysubstance use is universal, depression and anxiety are pervasive, and viral infections, neurological disorders and cognitive impairments are exceedingly common [2,5,6]. Unfortunately, the circumstances of marginalized persons in other communities across the world are similarly dire, with very high rates of psychopathological and medical afflictions (e.g., [7,8]).

Despite the ubiquity of psychopathology in marginalized populations, a reliable and valid instrument for its measurement has not been established. Although initially intended to identify positive, negative, and general aspects of psychopathology in schizophrenia, the Positive and Negative Syndrome Scale (PANSS; [9]) may prove to be suitable for this purpose. Indeed, in addition to assessing psychosis-related symptoms, the PANSS captures general psychopathology (including depression and anxiety) and cognitively-related features including “poor attention” and “difficulty in abstract thinking” [9,10]. Further, as opposed to many other instruments assessing psychopathology, the PANSS relies on observations and ratings making it appropriate for individuals with limited insight whom may be unable to provide reliable self-report.

The aim of the current study was to ascertain the PANSS factor structure in a heterogeneous sample of substance-dependent, marginally housed persons with prevalent psychiatric illnesses, including high rates of psychosis. This work is crucial to elucidating the structure of psychopathology in the above noted populations, and to determining the PANSS’s value in its measurement. Indeed, prior work has demonstrated that the PANSS can be used to evaluate psychiatric symptoms across different diagnostic groups, including bipolar disorder, post-traumatic stress disorder, and major depressive disorder [10–12]. Our approach was to: 1) identify a preliminary solution by conducting an exploratory factor analysis (EFA), 2) compute a confirmatory factor analyses (CFA) to replicate the EFA solution with a separate subsample, 3) replicate this model with data obtained 1-year later from the original participants, 4) conduct invariance analyses comparing Time 1 and Time 2 CFA models to assess temporal stability over one year, which to date has received limited attention [13–15], and 5) characterize the emergent factors by correlating them with clinical and functional variables.

## Materials and Methods

### Ethics statement

The Institutional Review Boards of participating universities (University of British Columbia and Simon Fraser University) approved all study procedures. Potential participants were provided with the consent form, and asked to read and consider its contents for at least 24 hours before signing. All participants subsequently provided written informed consent for participation, including written consent at enrollment to share relevant clinical results with their physician. Further, we developed and the Institutional Review Board approved a series of five questions that were asked at every assessment time point to ensure that the capacity for consent to participate remained valid, and was not complicated by a change in mental state. These questions included items assessing orientation and understanding of the goals of the study. Surrogate consent procedures (e.g., consent from next of kin or a legal representative) were not feasible for these marginalized persons.

### Study sample

Participants (n = 270) living in the DTES of Vancouver, Canada were enrolled in the study. Participants were recruited from SROHs (*n* = 266; see [2]) or through the neighborhood Downtown Community Court (*n* = 4), which specifically serves marginalized persons. The DTES is the poorest neighborhood in Canada [16]. Participants were 19+ years of age and fluent in English. Participants received honoraria for their time at each point of data collection.

### Assessments

Participants underwent comprehensive annual clinical and functional assessments [2,5], including the 30-item PANSS [9]. The PANSS has been used previously in unstably housed populations [17]. Psychiatrists diagnosed psychiatric and substance use disorders according to the Diagnostic and Statistical Manual of Mental Disorders (DSM-IV) [18]. The 77-item International Personality Disorder Examination Screening Questionnaire [19] was administered to identify those with likely personality disorders (PD) as defined by DSM-IV TR. Ratings for each of the 10 PD were dichotomized between those with three or less true/false items endorsed and those with more than three (suggestive of risk for that disorder). Depressive symptoms were assessed with the Beck Depression Inventory (BDI; [20]). A score of 14 or higher on the BDI has been used as a threshold for moderate to severe depression in marginally housed persons [21]. Everyday functioning was assessed with the Social and Occupational Functioning Assessment Scale (SOFAS; [22]).

### Statistical analysis

Participants were randomly assigned to either EFA (*n* = 100) or CFA subsamples (*n* = 170); 30 randomly identified EFA participants were also assigned to the CFA subsample in order to meet minimum sample requirements for both factor analytic procedures (i.e., EFA = 100, CFA = 200; [23–25]).

In keeping with previous psychometric research (e.g., [26]) EFA (performed using SPSS FACTOR) was performed using maximum likelihood method of factor extraction and varimax rotation [27]. The Kaiser-Guttman criterion was first applied to identify the number of eigenvalues greater than one, followed by the Cattell-Nelson-Gorsuch (CNG) scree test to examine the pattern of eigenvalue distribution.

Once a suitable EFA solution was identified, we tested this factor structure with CFA using a separate participant subsample in order to independently replicate the solution. In accord with the recommendations of O’Rourke and Hatcher [23], we computed and report three goodness-of-fit-indices. The Comparative Fit Index (CFI) is an *incremental index*, which represents the extent to which the hypothesized model is a better fit to data than a null model. The Standardized Root Mean Square Residual (SRMR) is an *absolute index*, which represents the standardized difference between observed and predicted correlations within the hypothesized model. Finally, the Root Mean Square Error of Approximation (RMSEA) is a *parsimony index*, which represents the extent to which the hypothesized model fits the data relative to the general population. Values greater than .94 for the CFI, and values less than 0.055 for the SRMR and the RMSEA, indicate good fit between models and data [23]. CFA was performed using the maximum likelihood method of parameter estimation.

One year after initial recruitment, the PANSS was re-administered to 201 participants (including participants from Time 1 EFA and CFA subsamples). Using these data, we planned to replicate the baseline CFA model, followed by performing invariance analyses to assess the temporal stability of the latent structure of the CFA model [28]. This was done by fixing parameter estimates for each item between models in succession. With each comparison, statistically significant change in the chi-square statistic indicated a significant between-model difference. CFA and invariance analyses were performed using the AMOS 20.0 statistical software using the maximum likelihood method of parameter estimation.

To help delineate the emergent factors, we inspected the associations between factor scores and external clinical and functional measures. Point biserial correlations were employed for dichotomous variables and Pearson correlations were used for continuous outcome measures.

## Results

### Participant characteristics and PANSS scores

As reported in Table 1, 7.0% of our sample met diagnostic criteria for schizophrenia. Seventy percent reported chronic (crack) cocaine use followed by heroin (36.2%) and cannabis (31.5%). Fewer methamphetamine (23%) and alcohol use disorders (19%) were diagnosed. PANSS ratings at both time points were slightly lower than first reported with a more homogeneous sample of those with schizophrenia (positive 18.20 (*SD* = 6.08), negative 21.01 (*SD* = 6.17), general psychopathology 37.74 (*SD* = 9.49); [9]).

**Table 1:** Sample characteristics.

Characteristic	% (#/total)	Mean (SD)	Range
**Demographics**			
Age (years)		43.80 (9.25)	25–67
Education (years)		10.21 (2.40)	2–16
Gender (Female)	22.2 (60/270)		
**PANSS Ratings (Time 1)**			
Positive		15.47 (6.01)	7–36
Negative		16.56 (6.28)	7–39
General Psychopathology		36.03 (8.50)	19–63
Total		68.03 (17.93)	33–132
**PANSS Ratings (Time 2)**^a^			
Positive		14.35 (5.98)	7–40
Negative		16.41 (5.96)	7–39
General Psychopathology		34.72 (7.64)	18–58
Total		65.47 (16.08)	34–126
**Psychiatric diagnosis**			
Schizophrenia	7.0 (19/270)		
Schizoaffective	5.9 (16/270)		
Psychosis not otherwise specified	13.0 (35/270)		
Bipolar I	5.6 (15/270)		
Major Depression	19.3 (52/270)		
**Substance Use Disorder**			
Alcohol	19.3 (52/270)		
Cannabis	31.5 (85/270)		
Cocaine	70.0 (189/270)		
Methamphetamine	23.0 (62/270)		
Heroin	36.2 (98/270)		
**Personality Diagnosis**^b^			
Paranoid	39.8 (102/256)		
Schizoid	23.4 (60/256)		
Schizotypal	37.9 (97/256)		
Antisocial	39.5 (101/256)		
Borderline	48.0 (123/256)		
Histrionic	37.9 (97/256)		
Narcissistic	38.7 (99/256)		
Avoidant	52.0 (133/256)		
Dependent	20.3 (52/256)		
Obsessive	36.7 (94/256)		
**Beck Depression Inventory**^c^		12.32 (11.15)	0–50
**SOFAS**		43.37 (13.10)	10–81

n = 270 unless otherwise specified; SOFAS = Social and Occupational Functioning Assessment Scale.

^a^n = 201.

^b^n = 256

^c^n = 255.

As indicated in Table 2, 68.4% of the study’s participants reported being homeless at least once. The majority of our sample is unemployed (91.4%) with a large proportion receiving income from welfare (i.e., BC Employment Assistance of 72.5%). The mean annual income is $11,202 with 97% of participants living in poverty (low income cut-off is $18, 421; [29]).

**Table 2:** Residence, employment, and income characteristics of the sample.

Characteristic	% (#/total)	Mean (SD)	Range
**Residence**			
Age moved to DTES^a^		33.0 (10.1)	12–59
Years on the DTES^a^		9.7 (4.9)	0.8–47.1
Ever homeless^a^	68.4 (182/266)		
Ever in jail or juvenile detention^b^	25.6 (69/270)		
**Employment**^c^			
Working at recruitment	8.6 (23/269)		
Unemployed	91.4 (246/269)		
Worked in the past year	12.7		
Worked 1 to 5 years ago	22.8		
Not worked for more than 5 years	53.4		
**Income Source at recruitment**^c^			
Long term disability	42.8 (115/269)		
Short term disability	5.6 (15/269)		
Welfare	72.5 (195/269)		
Pension	3.7 (10/269)		
Wages	8.6 (23/269)		
**Mean monthly income ($)**^d^		846 (417)	200–3,300
< 500	7.2 (19/264)		
500–1000	64.8 (171/264)		
1000–1500	23.9 (63/264)		
> 1500	4.2 (11/264)		
**Mean estimated annual income ($)**^d^		11,202 (4,898)	2,600–42,900

^a^n = 266.

^b^n = 270.

^c^n = 269.

^d^n = 264.

DTES = Downtown Eastside; for ‘Income Source’ 96 participants have two sources of income; therefore the total is greater than 100%. These include only legal sources of income. Estimated annual income was derived by multiplying monthly income by the thirteen pay periods.

### Exploratory Factor Analysis

EFA was performed in accord with prior PANSS research (e.g., [12]). The Kaiser-Meyer-Olkin (KMO) measure of sampling adequacy signified less than ideal interrelatedness as KMO = .68, indicating that less than 50% of variance is shared among PANSS items (i.e., .68 ^2^ = .46). The PANSS was a clinically-devised instrument, which may explain this relatively low percentage of explained variance.

The Kaiser-Guttman criterion suggested a 9-factor solution (i.e., 9 eigenvalues > 1.0). Yet as commonly noted [25], this criterion generally provides over-inclusive solutions. In contrast, the *10%+ variance criterion* suggests a more parsimonious, 2-factor solution as only eigenvalues 1 and 2 accounted for more than 10% of variance, λ_1_ = 7.12 (24%); λ_2_ = 3.31 (11%), respectively. A third factor accounted for 9% of variance just below the 10% threshold (λ_3_ = 2.61) whereas factors 4 and 5 accounted for just 7% (λ_4_ = 2.07) and 6% of variance (λ_5_ = 1.89), respectively.

The Cattell-Nelson-Gorsuch (CNG) scree test was next performed to examine the distribution of eigenvalues; discernible points where these values become level suggest the number of factors that best reflect the underlying latent structure. For this study, the scree test suggested that 3-, 4- and 5-factor PANSS models might be viable. As in this instance, ambiguous results can emerge because the CNG scree test is interpreted subjectively; scree test results can be self-evident in some studies (e.g., [26]) or quite ambiguous as we found. Combined, these findings suggest no discernible PANSS factor structure at this level of analysis as commonly reported EFA tests are inconsistent. Therefore, in order to further evaluate our PANSS data, computed models were examined next.

#### Computed models

A 5-factor structure was first tested as most, but not all, prior research suggests 5 factors, though with different item structures and factor names [30]. A 5-factor solution, however, was not psychometrically supported by our findings as all Factor 1 items cross-loaded on all other factors.

The 4-factor solution was similarly rejected as the majority of PANSS items cross-loaded on 2+ factors with only one item loading on the final factor (of 2 in total), suggesting a more parsimonious factor structure is likely. Generally, all factors should have 3+ items; in some instances, it is acceptable to have only two items on a final factor so long as the average per factor is 3+ for the overall model [23]. The 2-factor model exhibited a similar pattern of cross-loading; moreover, the item composition made interpreting these factors ambiguous; a 2-factor model was not clinically supported. A 3-factor model was last to be tested.

A 3-factor PANSS model seemed the most viable solution with this EFA subsample. The factor composition of this model is clinically discernible compared to other solutions, and all but 2 items load on these 3 factors (exceptions are G1, *somatic concerns*; and G6, *depression*). Provisionally, we have labeled these factors as F1) *Psychosis/Disorganized*, F2) *Negative Symptoms/Hostility*, and F3) *Insight/Awareness*.

### Confirmatory Factor Analyses, Time 1

**Fig 1:**
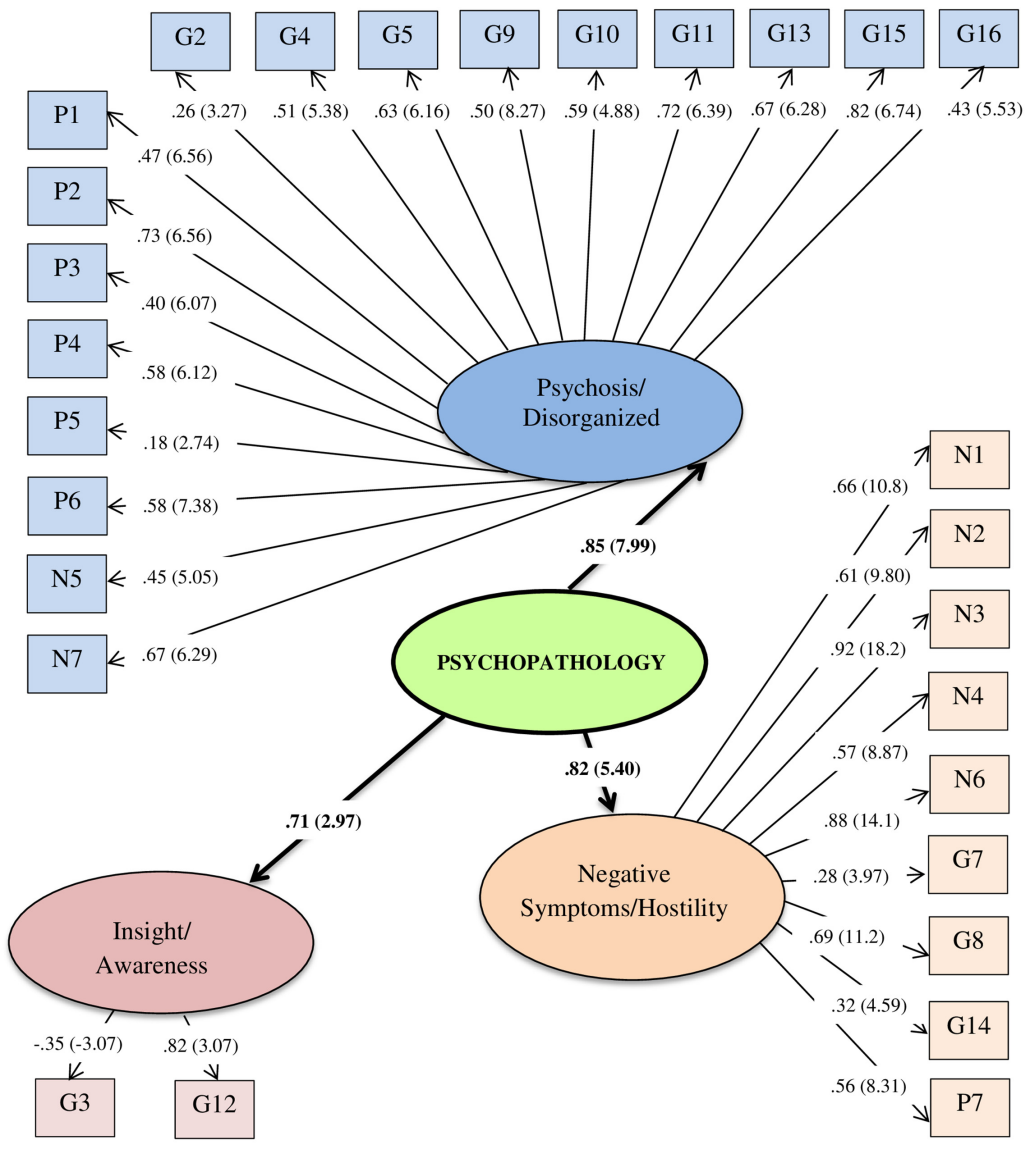
The three-factor model of psychopathology in our sample based on the Positive and Negative Syndrome Scale (PANSS). Maximum likelihood estimates, standardized solution and significance levels. Parenthetical numbers indicate significance levels for parameter estimates (statistically significant *t* values >❘1.96❘).

Next, we determined whether the 3-factor EFA model would be supported with a separate subsample (see section 2.4 for details). CFA was computed with each item loading on 1 of 3 hypothesized first-order factors; these 3 PANSS factors were, in turn, assumed to load upon a higher-order psychopathology latent construct. This model was supported. See Fig 1.

Each PANSS item loaded significantly upon its hypothesized factor (i.e., *t* values >❘1.96❘). This 3-factor model indicates effective fit of data, χ^2^(*df* = 265) = 366.51, *p* < .01. The SRMR for this model is adequate (SRMR < .09; SRMR = .070). In contrast, the CFI is within optimal limits (CFI > .94; CFI = .97) as is the RMSEA (RMSEA ≤ .050; RMSEA = .032); moreover, the full 90% confidence interval for the RMSEA statistic is within optimal parameters (.032 < RMSEA CL_90_ < .054). Statistical power for this 3-factor PANSS model is estimated at .99 (where α = .05).

### Confirmatory Factor Analyses, Time 2

The PANSS was re-administered to 201 participants a minimum of 10 months after initial assessment; the mean duration between points of measurement was 14.1 months (SD = 3.3 months; range = 11–30 months). CFA was again performed on Time 2 data to replicate the 3-factor PANSS model initially identified. Statistical power for this CFA model was estimated at .99.

With the exceptions of G2 (*anxiety*) and G4 (*tension*), each PANSS item again loads upon its respective first-order factor, and each of the three first-order factors loaded upon a higher-order psychopathology latent construct, χ^2^(*df* = 242) = 332.46, *p* < .01. The CFI is within optimal limits (CFI = .96) as is the RMSEA (RMSEA = .043) and the full 90% confidence interval for the RMSEA statistic (.031 < RMSEA CL_90_ < .054). Again, the SRMR for this model is adequate (SRMR = .073).

### Temporal analyses

Time 1 and Time 2 CFA analyses support the 3-factor PANSS model first identified. Yet replication of this proposed model does not necessarily indicate that the latent structure is stable (or invariant) over time. For instance, the item composition of factors can be consistent while the relative contribution to measurement of items upon their respective factor can significantly differ at separate points of measurement. To test temporal stability, we undertook invariance analyses as outlined by [31]. Analyses were conducted as a *partial test of measurement invariance* [32] as items G2 and G4 did not significantly load onto psychosis/disorganized factor at Time 2.

This was achieved by computing the 3-factor model with Time 1 and 2 data concurrently. Factor structures were first compared to determine if the 3-factor model mapping onto a second-order construct is consistent over time. This solution emerged as viable suggesting that the PANSS may best be measured by a three first-order factors mapping onto a higher-order psychopathology latent construct (χ^2^[*df* = 470] = 650.51, *ns*; CFI = .96; SRMR = .068; RMSEA = .031; .025 ≥ RMSEA CL_90_ ≥ .037). See Table 3.

**Table 3:** Summary specifications and temporal analyses of PANSS responses (Time 1 and 2).

PANSS Items		χ^2^	*df*	Δχ ^2^		Δ*df*	
**Baseline**		650.51	470				
**Psychosis/Disorganized**							
G5	posturing	652.22	471	1.71		1	
G10	disorient	652.56	472	.34		1	
N5	abstract	659.80	473	7.24 **		1	
P6	suspicion	679.03	474	19.23 **		1	
P1	delusions	685.66	475	6.63 *		1	
G9	thoughts	685.96	476	.30		1	
P2	disorganized	686.99	477	1.03		1	
P3	hallucinate	687.48	478	.48		1	
P4	excite	689.84	479	2.36		1	
N7	stereotyped	689.86	480	.02		1	
G15	preoccupied	691.47	481	1.61		1	
G11	attention	691.69	482	.22		1	
G13	volition	691.88	483	.19		1	
G16	avoidance	692.30	484	.42		1	
P5	grandiose	692.30	484	.42		1	
					41.79 **		14
**Negative Symptoms/Hostility**							
N3	rapport	695.91	485	3.60		1	
N6	spontaneity	697.04	486	1.14		1	
G8	cooperative	701.24	487	4.20 *		1	
G7	psychomotor	702.85	488	1.61		1	
N2	withdrawn	703.15	489	.34		1	
N1	blunted	735.17	490	32.02 **		1	
N4	apathy	736.94	491	1.77		1	
G14	impulse	737.28	492	1.15		1	
P7	hostile	738.43	492	1.49		1	
					46.12 **		8
**Insight/Awareness**							
G12	judgment	739.70	493	1.28		1	
G3	guilt	739.70	493	1.28		1	
					2.7		1
**Negative Symptoms/Hostility ← PP**		739.84	494	.14		1	
**Insight/Awareness ← PP**		739.84	495	.03		1	
**Psychosis/Disorganized ← PP**		739.84	495	.01		1	

PP = Psychopathology; four G Subscale items did not load significantly at either point of measurement (somatic concern, anxiety, tension, depression). Italicized items estimated values (initially fixed to 0 for scaling and statistical identification).

*p < .05

** *p* < .01.

We next compared the relative contribution to measurement of each item between models. This was accomplished by equating corresponding PANSS items in succession; significant change in the chi-square statistic indicates that the measurement properties of that item differ from Time 1 to Time 2. Three Factor 1 items differ between measurement points (N5, *difficulty in abstract thinking*; P6, s*uspiciousness/persecution*; and P1, *delusions*); the measurement properties of the remaining 12 items appear psychometrically equivalent. Two items from Factor 2 differ between points of measurement (G8, *uncooperativeness*; N1, *blunted affect)* whereas the remaining nine items appear invariant over time. The measurement properties of neither Factor 3 item significantly differ.

Although the psychometric properties of five PANSS items appear to differ between points of measurement, the relative contribution to measurement of each of the three first-order factors upon the higher order latent construct is equivalent from Time 1 to Time 2. Importantly, the underlying latent structure of this 3-factor model demonstrates temporal consistency. The differences in three Factor 1 and two Factor 2 items were insufficient to affect the psychometric properties of the 3-factor latent structure. Invariance analyses comparing Time 1 and Time 2 CFA models suggest considerable consistency between points of measurement. Factor analyses of cross-sectional PANSS data at recruitment were not definitive as preliminary analyses indicate that a 3-factor solution is the only viable factor solution. However, this 3-factor solution appears consistent over time.

### Clinical and functional associations

To further delineate the factors, we undertook correlational analyses (Table 4). Higher scores on Factor 1 (Psychosis/Disorganized) were strongly (i.e., *r* ≥ .50) associated with poor functioning (SOFAS ratings) and moderately (i.e., .20 < *r* < .50) associated with a diagnosis of schizophrenia, schizoaffective disorder, and psychosis not otherwise specified (PNOS). Higher scores on Factor 2 (Negative Symptoms/Hostility) were also associated strongly with poor functioning and moderately with a schizophrenia diagnosis. Factor 3 (Insight/Awareness) displayed a differential pattern as higher scores were significantly (albeit weakly; i.e., *r* ≤ .20) associated with lower levels of depressive symptoms (BDI ratings) and decreased incidence of the diagnosis of major depressive disorder (MDD). Factor 3 was also moderately associated with poor functioning and schizophrenia. The remaining significant associations were of weak magnitude.

**Table 4:** Clinical and functional associations of three factors.

Variable	Factor 1: Psychosis/ Disorganized	Factor 2: Negative Symptoms/Hostility	Factor 3: Insight/Awareness
Schizophrenia	.339***	.277***	.202**
Schizoaffective	.296***	.151*	.094
PNOS	.231***	.093	-.005
Bipolar I	.080	-.024	.049
MDD	-.034	.043	-.120*
Alcohol	-.082	-.091	-.074
Cannabis	.108	.097	.132*
Cocaine	.005	.081	.011
Methamphetamine	.139*	.053	.027
Heroin	-.075	.066	-.043
Paranoid^a^	.103	-.069	.021
Schizoid^a^	.002	.068	-.004
Schizotypal^a^	.131*	.083	.092
Antisocial^a^	.123*	.005	.036
BPD^a^	.120	-.054	-.123*
Histrionic^a^	.122	-.060	.019
Narcissistic^a^	.131*	-.024	.024
Avoidant^a^	.149*	.150*	-.010
Dependent^a^	.102	-.046	-.099
Obsessive^a^	.121	.022	.031
BDI^b^^,^ ^c^	.070	-.022	-.178**
SOFAS^c^	-.512***	-.515***	-.255***

PNOS = psychosis not otherwise specified; n = 270 unless otherwise specified; values represent point biserial correlations with dichotomous outcome variables unless otherwise specified.

^a^n = 256.

^b^n = 255.

^c^Pearson correlations.

*p < .05

**p < .01

***p < .001.

## Discussion

The results of the present study support the utility of the PANSS in marginally housed samples with high rates of substance use and psychiatric disorders. Indeed, our findings suggest that the PANSS captures psychopathology with three first order factors loading significantly upon this higher order latent factor, similar to findings reported by Van den Oord and colleagues [33]. Along with identifying and then replicating a 3-factor solution (after testing and rejecting the more commonly identified 5-factor solution, along with 2- and 4-factor models), we demonstrated that the model is temporally stable over a year. Longitudinal stability ensures that clinical instruments have the same measurement and scaling properties across time [34,35]. Few such studies have been conducted with the PANSS, yet those that exist have found stable and temporally invariant solutions [13–15]. We contend that the PANSS is appropriate for use with a broader range of psychiatric illnesses, including persons who are marginalized and suffering from polysubstance use disorders and other ailments. To our knowledge, this is the first study to evaluate the psychometric properties of the PANSS in a marginally housed sample with ubiquitous substance use and diverse psychiatric illnesses.

Inspection of our three-factor model revealed similarities with previous PANSS factor solutions. For instance, several of the highest loading items comprising F1 (Psychosis/Disorganized) correspond with similarly configured factors typically seen in schizophrenia, specifically: *disorganized* [12,33,36,37], and *cognitive* [38,39]. Our findings further indicate that F1 captures psychosis-related diagnoses based on moderate positive associations with schizophrenia and schizoaffective diagnoses and its associations with PNOS.

Likewise, the nine items entailing F2 (Negative Symptoms/Hostility) correspond to previously identified *Negative* factors or constructs [12,15,33,36–39]. Additionally, unique to this factor is the inclusion of P7, *hostility*. Elevated scores on this factor are associated moderately with schizophrenia and weakly with schizoaffective disorders. No significant associations were detected with depressive symptomatology or disorders (e.g., depression symptoms, or MDD), suggesting that the negative symptoms of the factor are aligned more closely with psychosis.

It is important to also address the distinctions between our factor model relative to previous solutions. The final factor (Insight/Awareness) to emerge was composed only of G12 (*lack of judgment and insight*) and G3 (*guilt feelings*; n.b. this factor negatively loaded onto F3, therefore high scores represent low levels of guilt feelings). High scores on this factor appear to represent individuals who have little insight or awareness into their psychiatric condition, life situation, and/or transgressions. The combination of these two items diverges from factors identified in schizophrenia-only samples, apparently reflecting a specific dimension of marginalized persons. Interestingly, relative to F1 and F2, this factor was associated with a distinct correlational pattern. Specifically, higher scores on this factor demonstrated a weak, yet significant, association with less depression symptoms. It is unclear whether this reflects a lack of insight or recognition of current depressive symptoms or truly lesser symptoms. Given this preliminary understanding of this factor’s associations, as well as the fact that only two items loaded on the factor, replication in other marginalized samples as well as additional clinical investigations of its nature is needed.

Lastly, two items did not load onto our 3-factor solution: G1 (*somatic concerns*) and G6 (*depression*). In other studies, these items tend to load onto a *Depression/Anxiety* factor (e.g., [15,36,38,39]. This suggests that depression and/or anxiety may manifest differently in psychopathologically heterogeneous samples and require additional clinical measures.

Despite the psychiatric heterogeneity of this marginally housed sample, the item composition of our first two factors corresponds with some previously identified solutions in schizophrenia without diagnoses of comorbid substance use disorders [15,33,37]. Yet most items load onto the first two larger factors, whereas in previously published research there is often distribution of items into 5 or more factors. Further, our factor structure differs from those commonly reported in that we found low loadings for items representing positive symptoms that characterize schizophrenia, namely delusions, hallucinations, and grandiosity. It is important to note though that even models derived from homogeneous schizophrenia samples often fail to adequately fit other schizophrenia samples [30], suggesting that the PANSS captures psychopathology, but does so differently across sample despite their similar diagnoses. This finding highlights the importance of validating the use of the PANSS in varied populations prior to using this as a measurement instrument of symptom dimensions and psychopathology.

### Limitations

Perhaps the most noteworthy limitation is that despite the stable factor structure we observed across time, less than half of item variance was explained meaning that PANSS items are not strongly correlated in this marginally housed sample with multiple psychiatric and substance use disorders. This contrasts previous PANSS factor structures that explain up to 84% of the variance in homogeneous samples [12]. As a result, there was greater correction for correlated error than is typical in CFA. It is likely that the extent of unexplained variance in these analyses can be attributed to the sample heterogeneity. Our participants differ across a host of psychiatric and substance use disorders that impact symptoms divergently, reducing the cohesiveness of item sets, increasing measurement error, and accounting for unexplained variance.

Additionally, we faced a sample size limitation in that to attain an appropriate sample size for the Time 1 CFA analysis (n = 200), we “borrowed” 30 participants that were used in the initial EFA. To minimize the impact, these participants were randomly selected and only made up 15% of the total CFA sample. Nonetheless, replication of the findings with a larger sized sample would be ideal, despite the fact that such samples are challenging to recruit and engage in longitudinal research.

Finally, beyond marginalized polysubstance abusing populations as reported here, the generalizability of the factor structure findings is apt to be narrow. This limitation is consistent with other empirical investigations that have not reached a consensus on the factor structure of the PANSS [30,33,36].

## Conclusion

We were able to identify and confirm a 3-factor PANSS model in our marginally housed sample of persons. We also demonstrated the stability of these symptom dimensions over a year duration. Along with its breadth of symptom coverage, these results suggest that the PANSS is an appropriate and valid clinical measure for more heterogeneous marginalized persons in which substance misuse is ubiquitous. Future research employing a Rasch or Mokken analysis in similar and divergent populations may further ascertain the psychometric properties of the PANSS vis-à-vis its clinical utility so that optimal measurement may be achieved.
